# SID/SIEDP expert consensus on optimizing clinical strategies for early detection and management of wolfram syndrome

**DOI:** 10.1007/s40618-024-02495-z

**Published:** 2024-11-11

**Authors:** Giulio Frontino, Maurizio Delvecchio, Sabrina Prudente, Valeria Daniela Sordi, Piero Barboni, Alessandra Di Giamberardino, Alessandra Rutigliano, Silvia Pellegrini, Amelia Caretto, Maria Lucia Cascavilla, Riccardo Bonfanti, Giuseppe D’Annunzio, Fortunato Lombardo, Lorenzo Piemonti

**Affiliations:** 1 Department of Pediatrics, Pediatric Diabetology Unit, Diabetes Research Institute, IRCCS San Raffaele Scientific Institute, Vita Salute San Raffaele University, Milan, Italy; 2 Department of Biotechnological and Applied Clinical Sciences, University of L’Aquila, L’Aquila, Italy; 3 Research Unit of Metabolic and Cardiovascular Diseases, Fondazione IRCCS Casa Sollievo della Sofferenza, San Giovanni Rotondo, Italy; 4 Diabetes Research Institute, IRCCS San Raffaele Hospital, Milan, Italy; 5 Vita-Salute San Raffaele University, Milan, Italy; 6 Department of Ophthalmology, IRCCS Ospedale San Raffaele, Milan, Italy; 7 Metabolic Disorder and Diabetes Unit, “Giovanni XXIII” Children Hospital, Bari, Italy; 8 Università Vita Salute San Raffaele, Milan, Italy; 9 Pediatric Clinic and Endocrinology, Regional Center for Pediatric Diabetes, IRCCS Istituto Giannina Gaslini, Genoa, Italy; 10 Department of Human Pathology in Adult and Developmental Age “Gaetano Barresi”, University of Messina, Messina, Italy

**Keywords:** Wolfram syndrome, Wolframine, Diabetes, Beta cells, Optic atrophy, Diabetes syndrome

## Abstract

Wolfram Syndrome (WFS) is a rare, multisystemic, degenerative disease leading to premature death. Clinical and genetic heterogeneity makes WFS diagnosis and management challenging. The Italian Society of Diabetes (SID) and the Italian Society for Pediatric Endocrinology and Diabetology (SIEDP) convened an expert panel of professional healthcare practitioners to provide up-to-date knowledge about the pathophysiology, clinical presentation and treatment of WFS, and recommendations for the earlydetection and optimal disease management. The consensus recommends the revision of diagnostic protocols to include genetic testing and comprehensive multidisciplinary evaluations to ensure accurate diagnosis of WFS, advocates for personalized management plans tailored to the unique needs of each patient, with an emphasis on exploring new potential drug therapies. A holistic care model that addresses the medical, psychological, and social challenges faced by patients with WFS and their families is strongly endorsed. The opinion underscores the importance of educating healthcare professionals about WFS to enhance early diagnosis and intervention, aiming to improve outcomes for patients through practical and evidence-based clinical strategies.

**Supplementary Information:**

The online version contains supplementary material available at 10.1007/s40618-024-02495-z.

## Introduction

Wolfram Syndrome (WFS) is a rare genetic neurodegenerative disease with juvenile onset, transmitted in an autosomal recessive mode. It is primarily characterized by diabetes mellitus (DM), optic atrophy (OA), diabetes insipidus (DI) and deafness (D), due to sensorineural hearing loss (SHL), collectively known as DIDMOAD [1]. Additional clinical features include other symptoms, such as urinary tract, endocrinological, renal, psychiatric, and neurological abnormalities. Urinary tract dysfunctions are more frequent than expected, prompting some to suggest that the acronym DIDMOADUD is better suited [2]. WFS was first reported in 1938 by Wolfram and Wagner who identified four of eight siblings with juvenile DM and optic nerve atrophy [3]. Wolfram syndrome is estimated to afflict about 1 in 160.000-770.000, but the prevalence varies across different regions. In North America it is estimated at 1 in 100.000, while in the UK it is reported to be 1 in 770.000 [4–6]. More recently, the estimated prevalence was 1 in 54.478 in the Messina district of north-eastern Sicily, 1 in 805.000 in Northern India, and 1 in 1.351.000 in Italy [7–9].

The gene responsible for WFS type 1 (WFS1), *WFS1*, is located on chromosome 4p16.1 and encodes the protein Wolframin, which plays a critical role in the regulation of the endoplasmic reticulum (ER)’s stability within pancreatic β cells and other tissues. Additionally, a second gene, *CISD2*, located on chromosome 4q22, has been identified in individuals with WFS type 2 (WFS2), which leads to early onset optic atrophy, diabetes mellitus, deafness, bleeding tendency and upper intestinal ulcers, a shortened lifespan, but notably not diabetes insipidus. The protein produced by *CISD2*, known as ERIS (ER intermembrane small protein), is also associated with the ER, although it does not directly interact with wolframin.

The prognosis of the WFS is primarily related to the severity of the neurological symptoms. Unfortunately, it is generally poor, with most patients experiencing premature death due to severe neurological disabilities such as bulbar dysfunction and organic brain syndrome. The median age at death is approximately 30 years (25–49 years) [6].

Diagnosing and managing WFS, given its genetic diversity and wide-ranging clinical manifestations, is complex. For patients and their healthcare providers, drawing connections between disparate symptoms to identify WFS as the underlying condition is not straightforward. Initial management often focuses on treating a single disease, commonly DM, with other associated conditions emerging gradually and subtly over time. This complexity leads to significant delays in obtaining a comprehensive diagnosis and appropriate care for individuals with WFS. Such delays can defer the initiation of suitable treatments, potentially worsening the disease’s progression and its associated conditions. The availability of specialized clinical and genetic resources for WFS varies significantly, influencing patient outcomes. A unified approach by healthcare and non-healthcare professionals could alleviate this, developing a dedicated clinical framework to support individuals with WFS and their families. WFS affects multiple body systems over time, necessitating a comprehensive range of multidisciplinary evaluations, screenings, and adaptive treatments. Effective guidelines for WFS require a collaborative, multidisciplinary approach.

This document targets healthcare professionals across all levels, aiming to streamline the diagnosis, treatment, and management of patients with WFS. It emphasizes the need for services spanning a broad array of specialties, crucial for comprehensive care (Table 1).

**Table 1:** The operating structures of the clinical centers of WFS

Department	Primary role	Interdisciplinary significance
Pediatrics	Central to the early identification and intervention of pediatric presentations	Serves as a foundational point for interdisciplinary coordination, ensuring early intervention and comprehensive care from childhood. It serves also as transition Coordinator, ensuring a smooth transition from pediatric to adult care and to avoid “dropout” from the health care system
Diabetology	Paramount in the therapeutic management of diabetes mellitus, an initial hallmark of WFS	Collaborates closely with endocrinology and internal medicine to manage metabolic aspects of WFS, providing a critical link in the multidisciplinary care chain
Endocrinology	Fundamental in tackling endocrine dysfunctions and their sequelae	Works in tandem with diabetology and genetics to address the hormonal imbalances, highlighting the importance of a cohesive approach to complex metabolic issues
Neurology and neurophysiology	Key in elucidating and managing the neurological and neuropsychological dimensions	Engages with psychology and radiology departments to offer a holistic approach to neurological care, emphasizing the importance of understanding the neuropsychological impact
Ophthalmology and neuro-ophthalmology	Essential for the detection and treatment of ocular pathologies, notably optic atrophy	Integrates with neurology to provide comprehensive care for visual and neurological manifestations, underscoring the need for specialized ophthalmic evaluation
Otolaryngology	Crucial for the auditory management prevalent among WFS individuals	Collaborates with audiology to enhance diagnostic and therapeutic strategies for hearing impairment, emphasizing interdisciplinary care for sensory deficits
Nephrology/urology	Significant in the management of renal and urological complications	Works alongside internal medicine to ensure a comprehensive approach to renal health and urinary system management, highlighting the complexity of WFS manifestations
Psychology	Indispensable for the psychological wellbeing of patients and familial units	Provides essential support across all departments, ensuring mental health be prioritized in the care plan, reflecting the holistic approach to patient care
Gastroenterology	Imperative for the symptomatic control of gastrointestinal manifestations	Collaborates with internal medicine to manage digestive health, illustrating the integrated care necessary for complex syndromic conditions
Internal medicine	Offers a holistic approach to the diverse symptomatology of WFS	Acts as the cornerstone for multidisciplinary care, coordinating with all specialties to ensure comprehensive management of the multifaceted aspects of WFS
Intensive care	May be required for managing acute, life-threatening complications	Provides critical support in life-threatening situations, highlighting the need for rapid, coordinated care among specialties
Medical genetics	Vital for the genetic delineation, diagnosis, and familial counseling	Collaborates across all specialties to guide the genetic understanding and counseling of WFS, ensuring a genetic perspective be integrated into the multidisciplinary approach
Neuroradiology and radiology	Critical for the advanced imaging and diagnostic elucidation of internal anomalies	Supports all departments by providing diagnostic clarity and aiding in the management of complex cases, highlighting the pivotal role of advanced imaging in the comprehensive assessment of WFS

Furthermore, coordination among general pediatricians, general practitioners, and health system authorities is paramount for a holistic care network. This multidisciplinary synergy is key to the effective management of WFS.

This consensus document aims to standardize care for patients with WFS, ensuring consistent access to diagnosis, treatment, and follow-up to enhance healthcare efficiency. It addresses the variability in clinical decisions caused by knowledge gaps and subjective treatment interpretations, striving for high quality, consistent care. The document, which results from an extensive review of the literature as well as of the available guidelines, focuses on managing WFS from childhood to adulthood, covering medical, psychosocial, and educational needs. It specifies a clinical pathway for health professionals dealing with pediatric and adult patients with clinical suspicion of WFS, aiming to ensure an early and correct diagnosis and apply management and treatment effectively. Finally, the document aims to improve care precision, enhance outcomes, and efficiently allocate resources, offering a systematic approach to diagnosis and treatment based on the more update scientific evidence and national and international guidelines. While expert-informed, it serves as a practical clinical tool, not replacing clinician judgment.

## Methods

The Italian Society of Diabetes (SID) and the Italian Society for Pediatric Endocrinology and Diabetology (SIEDP), considering new scientific evidence and the lack of updated guidelines, convened an Expert Panel to develop a national consensus on the diagnosis and management of WFS, addressing unmet needs and aiming to enhance current management and treatment strategies. Thirteen healthcare professionals, including endocrinologists, pediatricians, ophthalmologists, geneticists, psychologists, and laboratory scientists with expertise in WFS through patient care, clinical research, and peer-reviewed publications, were invited to join the Panel from leading Italian centers specializing in the diagnosis and treatment of WFS. Four of them (namely, G.F., M.D., S.P. and L.P.) joined the Steering Committee (SC) to lead the project and establish the aims and the topics of the consensus as well as the methodology to be followed to reach the agreement. The process unfolded in six phases: (1) an extensive literature review to identify key topics and unmet needs; (2) a kick-off meeting to organize the process and discuss content; (3) the implementation of a Delphi survey; (4) two rounds of online surveys to collect the Expert Panel’s opinions and achieve consensus; (5) analysis of the results by the SC and discussion of conclusions; and (6) paper writing.

In December 2023, SC members conducted a systematic review of the scientific literature using PubMed/Medline and gray literature (including scientific societies and patient associations). The search focused on articles published in English up to 2023, with search terms limited to titles and abstracts, including combinations such as:

1. “Wolfram Syndrome”.
2. “Wolfram Syndrome” AND (“management” OR “management goals” OR “guideline” OR “guidelines” OR “recommendation” OR “recommendations” OR “consensus”).
3. “Wolfram Syndrome” AND “treatment”.

Retrieved articles were screened according to title and abstract and considered if they were relevant for the definition of WFS clinical and molecular diagnosis, standard of care and management goals. Available Guidelines were also considered. At the end of the process (summarized in Supplemental Fig. 1), literature and guidelines revision allowed to consider 191 documents to accurately define the relevant topics and statements to be included in a Delphi survey. The survey, drafted by the SC members and including 12 main topics and 40 statements was then hosted online on a secure platform and submitted to all the panelists.

An evidence and consensus-based modified Delphi approach was used to reach consensus among the experts [10]. The experts rated each statement using a five-point Likert scale: 1 = strongly agree; 2 = agree; 3 = neutral; 4 = disagree; and 5 = strongly disagree. Consensus was a priori defined if > 75% of the experts agreed (1 e 2) or disagreed (4 e 5) on the Likert scale. Consensus was reached in two rounds of consultations held between February and April 2024. In the first round, the panelists answered the online survey with the possibility of adding their opinion and comments with open text. Information was then provided to the panelists by an anonymized summary of the results before commencing the following round. Following analysis of the answers provided, the statements were rephrased in the second round. All panelists completed the two rounds within the given timeframe, resulting in 100% participation. Round 1 resulted in consensus (> 75% agree or strongly agree) for 35 statements (87.5%). Round 2 (amended according to the comments of the panelists as well as revision of the statement in round 1 that did not reach consensus) resulted in consensus on all the statements.

All the responses were analyzed in aggregate only, while preserving the anonymity of the respondents. The results of the two rounds, presented in a descriptive form, were analyzed, interpreted and finally discussed jointly by the SC members to find common ground and provide useful recommendations to be included in the final document. Delphy survey showing the final obtained consensus results is available in Supplementary Table 1. Once the final draft was completed, it was sent to SID and SIEDP for final review and approval.

## Clinical presentation

WFS is a clinically and genetically heterogeneous condition. Two forms of WFS have been identified to date, both inherited in an autosomal-recessive manner. WFS type 1 (WFS1, OMIM #222300) is caused by biallelic mutations in the wolframin gene (*WFS1*) and represents the most frequent form. In particular, the human *WFS1* gene is located on chromosome 4p16, consists of eight exons, and encodes wolframin, a transmembrane glycoprotein of 890 amino acids in the ER [11]. WFS type 2 (WFS2, OMIM #604928) results from biallelic mutations in the *CISD2* gene, which are much rarer. Clinical features of WFS2 may include ulcers of the upper intestine, mucocutaneous bleeding, and defective platelet aggregation [12, 13]. In addition to the classic autosomal-recessive form of WFS, a subtype of WFS with autosomal-dominant inheritance called Wolfram-like syndrome (WFSL) was more recently identified. In this subtype, heterozygous mutations cause a less severe form of the classic disease, which is characterized by SHL with OA and/or impaired glucose homeostasis (OMIM #614296). A new autosomal-dominant hereditary syndrome has been reported in more recent studies, in which heterozygous mutations occurring *de novo* in the *WFS1* gene lead to a clinical condition more severe than that observed in classic WFS and characterized by neonatal diabetes, congenital cataracts/glaucoma, sensorineural deafness and hypotonia [14]. Heterozygous mutations in the *WFS1* gene can also lead to isolated and/or syndromic forms of autosomal-dominant inheritance, presenting with D, DM, and cataracts [15].

These specific forms of autosomal-dominant inheritance associated with heterozygous pathogenic variants of *WFS1* are collectively termed ‘*WFS1*-related disorders’, also known as ‘wolframinopathies’ (Table 2).

**Table 2:** Wolfram syndrome and *WFS1*-related disorders

	Denomination	Acronym	OMIM	Inheritance	Gene
Wolfram syndrome	Wolfram syndrome 1	WFS1	222,300	AR	*WFS1*
	Wolfram syndrome 2	WFS2	604,928	AR	*CISD2*
*WFS1-*related disorders	Wolfram-like syndrome	WFSL	614,296	AD	*WFS1*
	Genetic syndrome with neonatal or childhood onset diabetes, congenital sensorineural deafness, and congenital cataract	ND	ND	AD	*WFS1*
	Congenital Cataract 41	CTRCT41	116,400	AD	*WFS1*
	Autosomal-Dominant Deafness 6	DFNA6	600,965	AD	*WFS1*
	Non-insulin-dependent diabetes mellitus	*WFS1*-related diabetes	125,853	AD	*WFS1*

### Wolfram syndrome

**Fig. 1:**
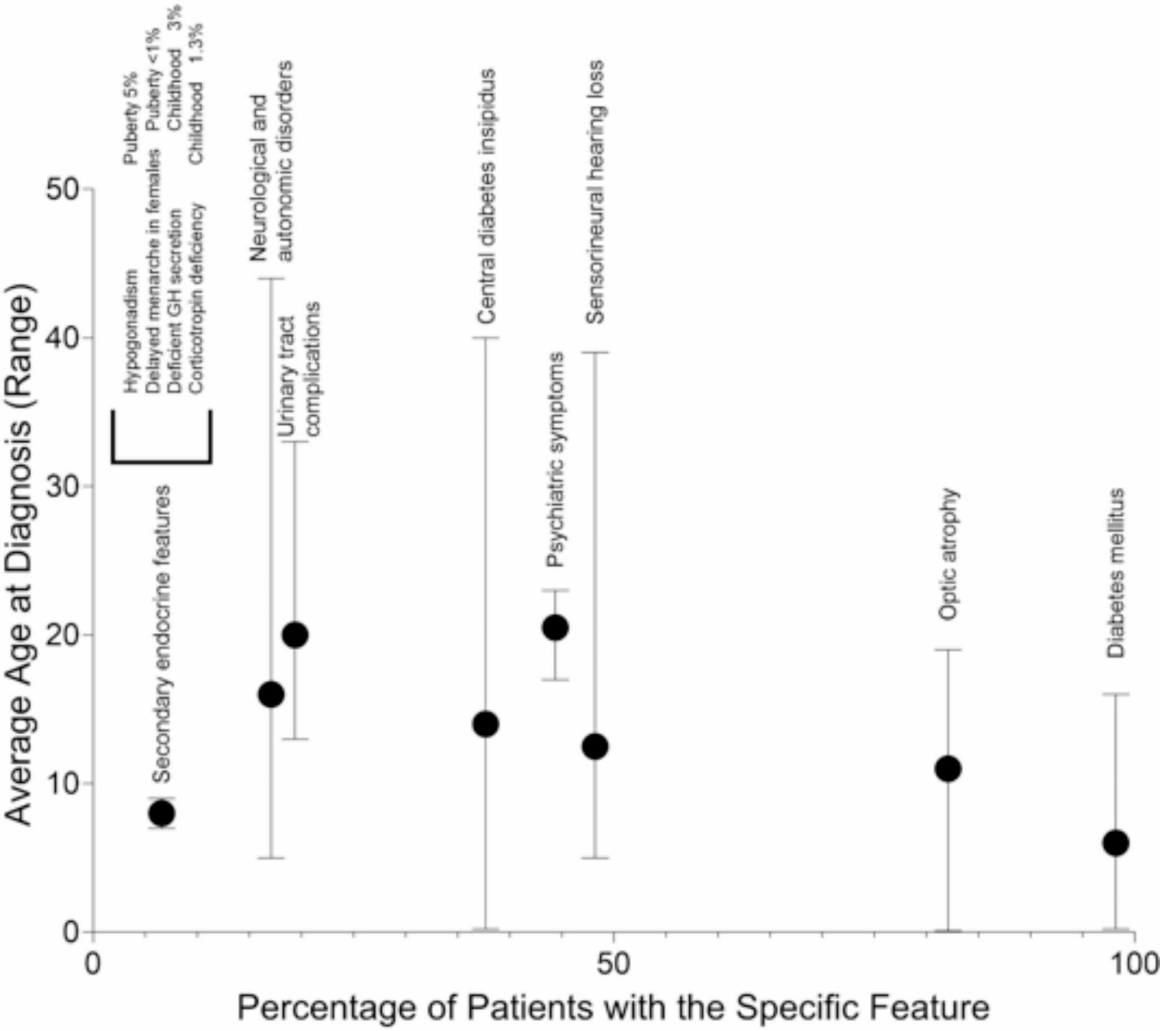
Clinical features of patients with Wolfram syndrome 1. Neurological and autonomic disorders include central apnea, ataxia, dysphagia, areflexia, epilepsy, decreased ability to taste and detect odors, headaches, orthostatic hypotension, hyperpyrexia, hypothermia, constipation, gastroparesis. Urinary tract complications include neurogenic bladder, bladder incontinence, urinary tract infections. Psychiatric symptoms include anxiety, panic attacks, depression, mood swings, sleep abnormalities, psychosis

The classic depiction of WFS largely stems from a foundational study by Barrett et al., 1995, involving 45 individuals from 29 families in the UK [6]. WFS is primarily identified by the early onset of DM and OA, typically before the age of 16. The study revealed also that 64% of individuals manifested SHL by age 20. Additionally, it was found that 60% displayed at least one of several clinical symptoms: cerebellar ataxia, peripheral neuropathy, intellectual disability, dementia, psychiatric disorders, and urinary tract dysfunction, with the subjects having an average age of 16 years (ranging from 5 to 32 years). Although other organs might be implicated, comprehensive clinical evaluations covering the full extent of the syndrome’s impact have been limited. Thus, the intricate dynamics of multi-organ involvement in WFS and its complete natural history remain largely unexplored. Table 3 presents a summarized overview of the primary clinical characteristics associated with WFS while Fig. 1 summarizes the frequency and the range of age at onset of the most important clinical characteristics of WFS.

**Table 3:** Clinical features of WFS

Feature	Detailed description
Diabetes Mellitus (DM)	Typically, the initial manifestation, DM in WFS usually presents during the first decade of life, with an age range of onset from less than 1 year to 17 years. Nearly all diagnosed individuals require insulin therapy to manage their condition. Unlike autoimmune diabetes mellitus, DM in WFS tends to follow a milder course with a notably lower incidence of microvascular complications
Optic *Atrophy* (OA)	A universal feature among those with WFS, OA leads to a progressive loss of color vision and peripheral vision. The condition generally manifests within the first ten years of life, gradually progressing to severe visual impairment over years. Visual acuity may deteriorate to a level where the affected individual sees at 3 m what a person without the disease can see at 60 m. In rare cases, genetic variants of WFS can result in isolated OA with an autosomal recessive inheritance pattern
Sensorineural Hearing Loss (SHL)	This condition affects approximately 66% of individuals with WFS, presenting a range of severity from congenital deafness to mild hearing loss that begins in adolescence and worsens with age. The average age of onset is approximately 12.5 years. Studies have confirmed a preference for high-frequency sounds to be affected and noted the condition’s slowly progressive nature, with no significant sex differences in the degree of hearing loss observed
Neurological dysfunctions	At least 62% of individuals experience neurological symptoms, with a median age of onset at 15 years (ranging from 5 to 44 years). The data on specific neurological abnormalities is limited, but current observations indicate that symptomatic neurological manifestations often emerge by the fourth decade, with initial onset typically between the first and second decades. These manifestations are progressive, primarily involving general brain atrophy, and affect regions such as the brainstem, cerebellum, and cranial nerves. Ataxia and central apnea are among the severe manifestations observed
Psychiatric Illnesses & dementia	There is a significantly increased risk of psychiatric conditions, including suicidal behavior, among individuals with WFS. While intellectual disability is not commonly associated with WFS, dementia has been observed as part of a broader neurodegenerative disorder in some cases
Brain MRI findings	A study by Samara et al. in 2020 reported various MRI findings in individuals with WFS, including reduced or absent “bright” signal of the neurohypophysis, optic nerve atrophy, T2-weighted white matter hyperintensities, and cerebellar atrophy. These findings underscore the progressive nature of the neurological involvement in WFS
Diabetes Insipidus	Central origin diabetes insipidus has been reported in 72% of individuals affected by WFS, with a mean age of onset at 15.5 years. The broad range in age of onset may reflect delays in accurate diagnosis
Hypogonadism & fertility	Hypogonadism is more common and severe in males than females, with a consequent reduction in fertility more pronounced in males. The underlying causes of hypogonadism in WFS, whether hypogonadotropic or hypergonadotropic, remain unclear
Central hypothyroidism	Although central hypothyroidism is described in individuals with WFS, the frequency of its occurrence is not well established
growth	Some individuals with WFS experience growth retardation, although most achieve an adult height within the normal range. Cases of growth hormone deficiency have been reported, highlighting another aspect of the syndrome’s complexity
Urinary tract problems	Between 60% and 90% of patients with WFS experience urinary tract issues, including hydroureter, urinary incontinence, and recurrent infections, indicative of a neurogenic bladder. Urodynamic examinations have identified findings consistent with bladder atony and progression to megacystis, with potential for acute urinary outflow obstruction
Gastrointestinal dysfunction	Up to 25% of individuals with WFS report gastrointestinal issues, such as constipation, chronic diarrhea, and other intestinal dysfunctions. This adds another layer to the diverse symptomatology seen in WFS

#### Clinical aspects

Insulin-dependent diabetes mellitus (DM), of non-autoimmune origin and caused by the loss of β-cells, is usually present and the first sign of the disease. However, some case reports have documented β-cell autoimmunity in adult patients with WFS [16]. Patients with WFS1 typically have better HbA1c levels, lower insulin requirements, and fewer microangiopathic complications compared to those with type 1 DM [17, 18]. DI originates centrally and is usually diagnosed in the second decade of life. OA is typically diagnosed during the first decade of life. Ophthalmological findings include severe axonal loss and demyelination of the optic nerves, as well as the chiasma tracts. The development of OA is associated with the impact of *WFS1* gene mutations on retinal ganglion cells (RGCs). The damage starts with the axons of RGC; over time, this axonal damage can progress, resulting in the loss of RGC bodies [19]. Other less common ophthalmological findings related to WFS include diabetic retinopathy, retinal changes, cataracts, glaucoma, nystagmus, and abnormal pupillary light reflexes [20–22]. Sensorineural deafness is diagnosed around age 16 in 60% of cases, marked by significant shifts in auditory thresholds at medium to high frequencies. Audiometric features consist of severe auditory threshold shift, more evident for the medium/high frequencies. Wolframin is expressed in inner ear cells and its effects have been verified in both the cochlear nerve and the organ of Corti. Phenotypic manifestations are not constant, suggesting that SHL could be a consequence of a dysfunction in different sites of auditory pathways [23]. Individuals with WFS have been observed to experience various early endocrine dysfunctions, in addition to DI. These include primary and secondary hypogonadism, more common in males, while females may also experience menstrual irregularities and delayed puberty [24]. Anterior pituitary hypofunction is of hypothalamic origin and can lead to growth hormone deficiencies and impaired corticotropin secretion. Monitoring growth velocity, pubertal development, and considering steroid supplementation during periods of stress or illness are crucial. Urinary tract dysfunctions, usually observed in adulthood, have recently been reported in younger individuals with WFS as well. These include ureterohydronephrosis due to bladder dysfunction, as confirmed by urodynamic testing, and autonomic neuropathy. Both high-capacity atonic bladders and high-pressure, low-capacity bladders have been reported. Delayed diagnosis and treatment of urinary tract issues can increase the risk of severe infections and acute and chronic renal failure [25]. WFS is also associated with central nervous system abnormalities like anosmia, ataxia, seizures, nystagmus, gaze palsies, dysarthria, dysphagia, psychiatric disturbances, cognitive impairment, and others. Severe neurological impairment typically involves ataxia, dysarthria, neurogenic bladder, dysphagia, dementia, and gait problems. Despite the potentially life-threatening neurological complications associated with WFS, the complete extent of neurological damage remains unclear and is determined through clinical assessments and post-mortem analyses [26]. Recent studies indicate that early brain vulnerability in WFS may be associated with decreased intracranial volume, which impacts the integrity of both gray and white matter in specific brain regions, including the brainstem, cerebellum, and optic radiation. This vulnerability may be due to ER stress caused by *WFS1* mutations, impacting brain development initially and leading to neurodegenerative effects later [27]. These abnormalities were also observed in younger patients who exhibited few clinical symptoms and lacked an age-dependent progression. It is hypothesized that endoplasmic reticulum (ER) stress caused by WFS1 gene mutations impairs early brain development, leading to subsequent neurodegenerative effects [28].

### WFS1-related disorders

Data about prevalence of *WFS1*-related disorders are not currently available.

#### Wolfram-Like syndrome (WFSL)

WFSL is less common than classical WFS. At least 14 families with WFSL have been reported to date [14, 29–33]. Most individuals with WSFL present with isolated OA and congenital deafness. Some families may also exhibit DM, either as isolated condition or in combination with OA and D.

A recent meta-analysis involving 86 patients with WFSL revealed that a range of clinical manifestations—including OA, DM, HI, DI, as well as other endocrine, neurological, urological, and psychiatric symptoms—can present in various combinations. The most frequently observed phenotype combines OA and HI, seen in 47% of patients, while DM is present in 44%. Cataracts are also a common ophthalmic feature in WFSL. Typically, HI is the initial manifestation, usually appearing in the first decade, followed by DM a few years later and OA in the second decade [34]. While published follow-up data are limited, the available data suggest a non-progressive clinical condition with a milder phenotype compared to classical WFS. Additionally, affected individuals do not appear to develop progressive neurodegeneration, although further neuroradiological studies are still lacking.

Notably, some *WFS1* mutations have been identified in a compound heterozygous state in patients with classical WFS and in a single heterozygous state in those exhibiting the WFLS phenotype [35–38]. These findings raise questions about the relationship between WFS and WFLS. It remains unclear whether they represent distinct disease entities with different inheritance patterns or if they fall within the same condition, which may segregate in a recessive manner but exhibit reduced penetrance in heterozygous carriers. This ambiguity complicates genetic counseling and the management of asymptomatic individuals who carry heterozygous *WFS1* variants.

#### Genetic syndrome with neonatal or childhood-onset DM, congenital SHL and congenital cataracts

Recently, five probands were reported to have *de novo* heterozygous mutations in the *WFS1* gene, presenting with a severe form of disease characterized by neonatal or childhood-onset DM, congenital cataract, and SHL [14]. Notably, this new syndrome, due to specific dominantly acting mutations in *WFS1* which through protein aggregation actively induce ER stress, shows a discrete pathophysiology and differs genetically and clinically from classical WFS often resulting from complete absence of WFS1 protein caused by recessive null mutations.

## Diagnosis

**Fig. 2:**
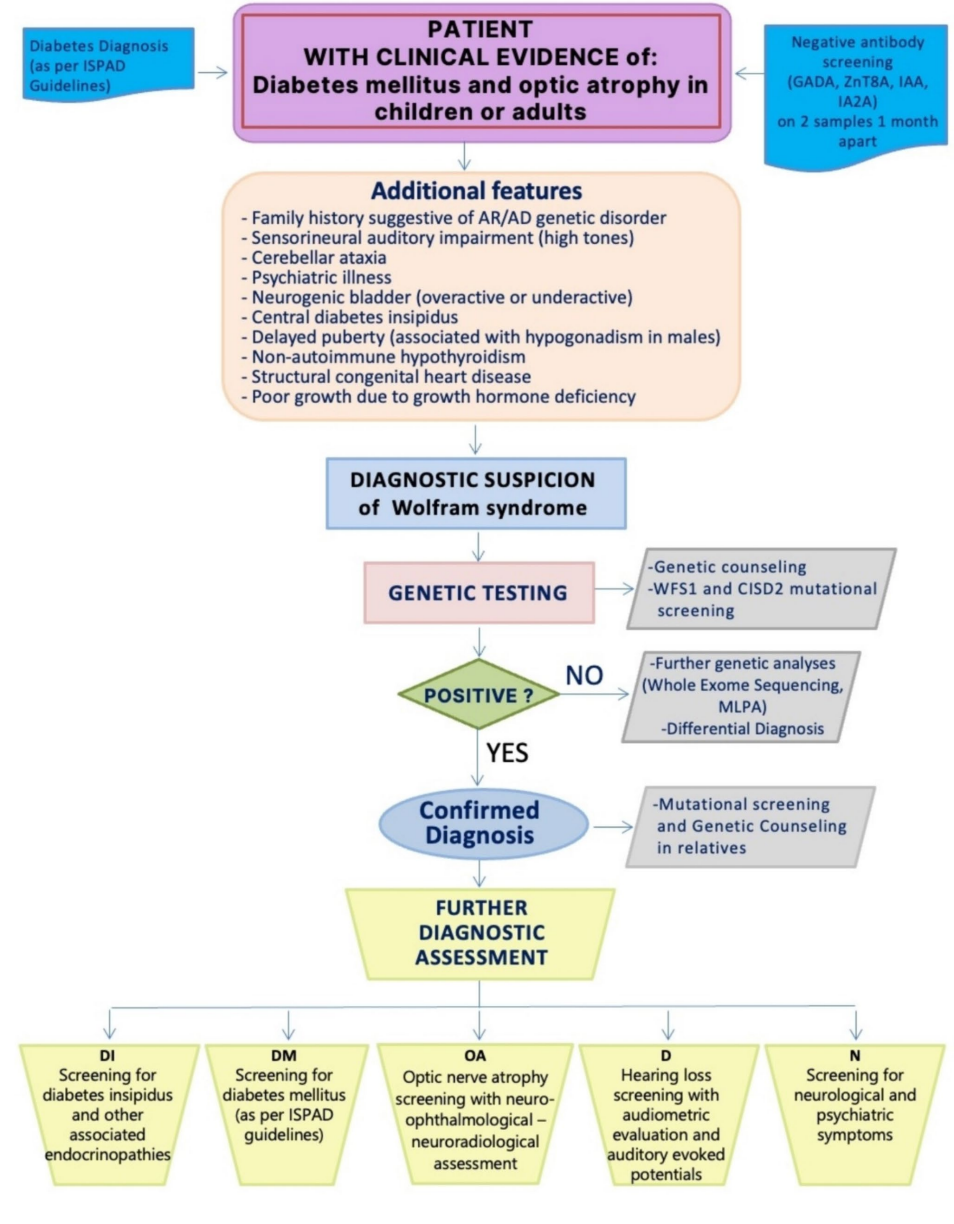
Diagnostic assessment roadmap

WFS is primarily determined by pathogenic or likely pathogenic biallelic variants (defined according to ACMG classification criteria) in (i) the *WFS1* gene (NM_006005. 3) that encodes wolframin, an ER membrane glycoprotein crucial for Ca^2+^ homeostasis and regulation of the ER stress response or in (ii) the *CISD2* gene (NM_001008388.5) that encodes a protein located in the ER and mitochondrial membrane [39–41]. Classical WFS follows an autosomal recessive transmission pattern, where pathogenic or likely pathogenic variants in *WFS1* and *CISD2* genes may be present in probands either in homozygosity (often observed in probands from families with consanguinity) or in compound heterozygosity. In addition to early onset of DM and OA and possibly suggestive family history, WFS may manifest with a variety of additional clinical features previously reported [6, 42, 43]. The absence of a known family history does not preclude the diagnosis. The diagnostic assessment roadmap is displayed in Fig. 2.

### Genetic testing

While medical and family history, along with clinical findings, are crucial for diagnosis of WFS, genetic testing is essential in confirming the clinical diagnosis. Therefore, patients with a clinical suspicion of WFS should be referred for genetic testing to detect mutations in the *WFS1* or in the *CISD2* genes. Molecular analysis of these two genes can be performed using massive parallel Next Generation Sequencing (NGS). This can be achieved either through targeted panels containing the specific genes of interest or by whole exome or genome sequencing (WES, WGS).

It is strongly recommended that molecular analysis must be conducted at specialized diagnostic centers. Given the progressive nature of the disease and its significant prognostic implications, the results of genetic testing should be communicated within a comprehensive genetic counseling session. Ideally, a multidisciplinary team, which includes a geneticist along with other clinical specialists involved in the disease management (i.e. endocrinologists, ophthalmologists, audiologists, neurologists and psychologists), should facilitate this session. It is advisable to extend molecular analysis to the parents and first-degree relatives of probands whenever possible. In clinically diagnosed patients, genetic testing revealing pathogenic or likely pathogenic biallelic variants in the *WFS1* or *CISD2* genes confirms the clinical diagnosis of the classic recessive form of the disease.

Although in 90% of patients clinically diagnosed with WFS, both pathogenic variants in *WFS1* or *CISD2* are detected by genetic testing, in some patients only one pathogenic variant is identified [44]. This points to: (i) the lack of detection of a second mutation (if located in the promoter or 5′- or 3′-untranslated regions of WFS1 and CISD2 mRNA, which are not routinely included in the mutational screening or due to technical pitfalls), (ii) the existence of dominant mutations, or (iii) the presence of mutations in another gene. In a small percentage of patients with a clinical diagnosis of WFS no variants of clinical significance are identified [42]. Pathogenic variants in the coding sequence of the *WFS1* gene encompass deletions, insertions, and single nucleotide changes that result in frameshift or non-frameshift mutations, as well missense and nonsense mutations [45]. The mutations identified as causing WFS1 (358 classified as pathogenic/likely pathogenic in *WFS1* gene, LOVD database https://databases.lovd.nl/shared/transcripts/00023835, update to 23 march 2024) are distributed along the entire length of the coding sequence [46]. However, they are predominantly concentrated in the longest exon, i.e. exon 8. Although some mutations have been found in more than one family, most families have ‘private’ mutations [42, 45]. Conversely, the few mutations to date identified in the *CISD2* gene as causing WFS2 encompass deletions and missense amino acid changes [47]. For the variants classification, laboratories should consider using the 5-category system proposed and validated by the American College of Medical Genetics and Genomics (ACMG) in collaboration with the Association for Molecular Pathology (AMP) [40]. This system defines each variant as follows:

- Pathogenetic (causative; class 5).
- Likely pathogenic (causative; class 4).
- Of uncertain clinical significance (“VUS”; class 3).
- Likely benign (class 2).
- Benign (class 1).

The process of classifying variants according to the ACMG-AMP guidelines relies on different types of evidence, which may evolve over time as new data become available in the literature. Consequently, previous variant classifications are subject to potential changes based on updated evidence. Variants previously assigned to a certain class may be upgraded or downgraded based on new findings and interpretations [48]. According to this system, the classification of a variant should be considered as definitive when it is pathogenic (Class 5) or benign (Class 1). Indeed, it is believed that only in very few cases there will be a change in classification because of the accumulation of new evidence, although examples of such changes are known. On the other hand, a change in classification over time can be expected for Class 4 (likely pathogenic) and especially for Class 3 (VUS). Particular attention must be paid to class 3 variants, classified as VUS. The detection of such variants, which does not immediately allow them to be considered either as benign or deleterious, introduces uncertainty for the patient and potentially for the physician, because a VUS cannot (and should not) be used in clinical decision-making. Importantly, the classification of a variant as a VUS is often achieved when it is newly identified. When such variants are found, a systematic review process should be implemented, conducted at regular intervals (e.g. annually), to assess the availability of new evidence useful for reclassification. Reclassification requests should be initiated by the geneticist and/or the clinical specialist responsible for the patient’s management and addressed to the designated committee or laboratory responsible for variant interpretation.

### Assessment of relatives at risk

In families of probands with identified mutations in the *WFS1* or *CISD2* gene, genetic counseling should be conducted, and genetic testing should be offered to parents and siblings (especially thosewho are younger and apparently still asymptomatic) as well as to other available relatives. This approach aims to identify early mutation carriers and individuals potentially at risk of disease, thereby enabling clinical and early initiation of treatment for the first manifestations of WFS, WFSL or *WFS1*-related disorders, that may coexist within the same family.

### Genetic counseling

#### Pre-test counseling

Pre-test counseling is a critical step in the genetic testing process for WFS, ensuring that the individual and, when applicable, their family, are fully informed about the following aspects:

1. Genetic Inheritance of WFS: Discussing the autosomal recessive nature of WFS to help families understand potential hereditary implications.
2. DNA Sample Collection: Providing clear instructions on how to collect a DNA sample effectively ensures the accuracy of test results.
3. Informed consent: Outlining the need for informed consent according to the testing company’s guidelines, highlighting the significance of content comprehension and privacy considerations.

4. Test Appropriateness: Assessing the likelihood that the selected genetic test aligns with the clinical suspicion of WFS.
5. Test Sensitivity and Specificity: Explaining the test’s accuracy, including its ability to detect *WFS1/CISD2*-related genetic variants accurately.

#### Post-test counseling

The patient (and, if a minor, their parents) have the right to be informed about the results, which must be formulated in a written report signed by the head of the laboratory responsible for their generation.

Upon receiving a positive test result, it is crucial to address several key areas in post-test counseling:

1. Interpretation of Results: Explaining the meaning of identified variants and their implications for the patient’s health and management.
2. Disease-Specific Exemption Code: Discussing the assignment of a disease-specific exemption code, considering some patients’ preferences not to be officially recognized as having the condition.
3. Treatment Options: Informing patients about existing and potential therapeutic interventions.
4. Clinical Monitoring: Advising on the necessity of regular clinical follow-up to manage and monitor the condition effectively.
5. Privacy and Confidentiality: Ensuring patients understand who owns their genetic information and the level of confidentiality with which it will be treated.

### Genotype-phenotype correlation

Thanks to a recent meta-analysis of case report data published and included in a systematic review by de Heredia et al. along with patient data from the Washington University’s International Registry of WFS and *WFS1*-related Disorders, Lee et al. in a recent study were able to assess genotype-phenotype correlations [42, 45]. This study allowed to identify a significant correlation between pathogenic variant characteristics and disease severity. The research revealed that the severity of the phenotype occurring in WFS, and *WFS1*-related disorders correlates with the number of pathogenic genotypic variants in the *WFS1* or *CISD2* gene present in patients (two for the classical WFS recessively inherited or one for *WFS1*-related disorders presenting with an autosomal-dominant inheritance) and their impact on the coding sequence or the position of the variants within the wolframin trans-membrane domain.

The study showed that:

1. A greater number of pathogenic genotypic variants in *WFS1 or CISD2* gene correlates with an earlier onset and more severe presentation of the syndrome.
2. The presence of nonsense and frameshift variants correlates with more severe phenotypic presentations than the presence of missense variants and is correlated with an earlier onset of DM and OA in patients carrying two nonsense/frameshift variants than in those carrying none or 1 nonsense/frameshift variant.
3. The number of transmembrane in-frame variants present in patients (one or two) determines a statistically significant dose effect on the age of onset of diabetes mellitus and optic atrophy.

Notably, heterozygous variants of *WFS1* gene can result in one of the *WFS1*-related disorders listed in Table 2. Generally, heterozygous missense variants in *WFS1* result in clinical phenotypes that are milder compared to classical WFS and do not typically exhibit a decrease in life expectancy [15].

However, an exception to this observation has recently been documented in a subgroup of patients.

Despite having heterozygous missense variants in *WFS1* gene, these patients exhibited a syndromic and more severe phenotype than classical WFS. They presented with onset of DM within the first year of life, along with hypotonia, congenital SHL and congenital cataracts [14]. Importantly, the variants found in these probands were not inherited from either parent but arose *de novo* in each case. Finally, WFS and WFSL can coexist in the same family thus supporting the notion that heterozygous carriers are not necessarily healthy individuals as usually they are considered [34].

## Differential diagnosis

In WFS, the differential diagnosis between WFS1 and WFS2 can be clinically established based on the presence of signs and symptoms observed in the proband, and molecular confirmation can be achieved through genetic testing of the *WFS1* and *CISD2* genes. Given that some signs and symptoms of WFS can overlap with those of other genetic conditions, special attention must be paid to the differential diagnosis of WFS from conditions featuring syndromic SHL, along with ocular defects and neurological abnormalities, with or without DM (Table 4).

**Table 4:** Differential diagnosis for WFS and *WFS1*-related disorders

Condition to be differentially diagnosis	Gene(s)/genetic mechanism	MOI	Endocrine abnormalities	Eye disorders	Hearing loss	Neurological abnormalities
Alstrom syndrome	ALMS1	AR	Insulin resistance/DM type 2 typically begins in the second decade. Other abnormalities include hypogonadotropic hypogonadism in males, polycystic ovaries in females, and hypothyroidism. Common obesity can lead to non-alcoholic hepatic steatosis	Progressive visual impairment due to dystrophy of the rods and cones starts between birth and 15 months; often results in no light perception by age 20	Sensorineural hearing loss usually starts in the first decade, potentially becoming moderate to severe (40–70 dB) by the end of the first or second decade	Detrusorurethral dyssynergy in females by the end of the second decade
Bardet-biedl syndrome	Multiple BBS genes, MKKS, MKS1, TTC8	AR	Insulin resistance/DM type 2 appears in adolescence or adulthood; common obesity; male hypogonadotropic hypogonadism	Rod and cone dystrophy leading to night blindness by age 7–8 years; average age of legal blindness is 15.5 years	50% of adults develop sensorineural hearing loss (SNHL) detectable only by audiometry	Significant learning difficulties in the majority; severe IQ test impairment in some
Myotonic Dystrophy Type 1 (DM1)	DMPK	AD	DM is common	Cataracts in mild and classic forms	No data available	Mild myotonia in mild DM1; muscle weakness/atrophy and myotonia in classic DM1
Friedreich’s Ataxia	FXN	AR	30% have DM	Optic nerve atrophy occurs in ~ 25%, often asymptomatic; progressive decrease in contrast acuity	SNHL in 13% of cases	Slowly progressive ataxia; dysarthria, muscle weakness, spasticity, scoliosis, bladder dysfunction, sensory loss
Kearns-Sayre Syndrome	mtDNA deletion	Mat	DM, hypoparathyroidism, growth hormone deficiency	Pigmentary retinopathy and progressive external ophthalmoplegia before age 20	Hearing loss in some cases	Cerebellar ataxia; intellectual impairment
Thiamine-sensitive megaloblastic anaemia syndrome	SLC19A2	AR	DM from infancy to adolescence; may be thiamine sensitive	Optic atrophy mentioned in case reports	Progressive SNHL, often detected in young children, not prevented by thiamine	Neurological deficits including stroke and epilepsy reported in early childhood in 27% of cases
Optic atrophy type 1	OPA1	AD	No DM	Bilateral, symmetrical optic nerve pallor with variable visual impairment starting ages 4–6; visual field and color vision defects	Auditory neuropathy leading to SNHL, ranging from severe and congenital to subclinical	~ 20% exhibit additional neurological signs
Charcot-Marie-tooth X Type 5 neuropathy	PRPS1	XL	No DM	Optic neuropathy in males with visual impairment onset between 7–20 years	Bilateral early-onset profound SNHL in males	Peripheral neuropathy in males, onset 5–12 years
Deafness-dystonia-optic neuropathy syndrome	TIMM8A	XL	No DM	Progressive decrease in visual acuity due to optic atrophy starting around 20 years in males	SNHL in early childhood in males; mild hearing issues in females	Slowly progressive dystonia or ataxia starting in the second decade; dementia and psychiatric symptoms can appear in childhood and progress. Females may experience focal dystonia

## Treatment

### Pharmacological treatments

Currently, there is no specific treatment for WFS. Each disorder associated with the syndrome can be treated, using hormone replacement therapies or palliative care (Table 5). Although these approaches do not address the underlying cause of the condition, careful clinical monitoring and supportive care can help mitigate its debilitating effects.

**Table 5:** Management of Wolfram syndrome manifestations

Manifestation	Management strategy	Therapeutic options
Insulin-dependent Diabetes Mellitus (DM)	Follow standard clinical practices for managing insulin-dependent DM	- Advanced Hybrid Closed Loop Systems: Adjust insulin delivery based on continuous glucose monitoring - Multi-injection Insulin Therapy: Multiple daily insulin injections based on the patient’s lifestyle and glucose monitoring
Optic nerve and retinal atrophy	No proven treatment for optic nerve atrophy; management recommendations based on neuro-ophthalmologist’s advice	Coenzyme Q Derivatives (Idebenone): Proposed to improve mitochondrial function, though effectiveness specific to optic nerve atrophy in WFS is not fully established
Sensorineural Hearing Loss (SHL)	No direct treatment to reverse SHL; intervention based on degree of hearing loss	Cochlear Implant Placement: Considered for individuals with significant hearing loss, usually carrying homozygous mutations, to improve hearing capability
Others Endocrinopathies	Treat with hormone replacement therapy, tailored to the specific hormonal deficiency	Hormone replacement therapy type varies depending on the affected endocrine system (e.g., thyroid hormone, testosterone, etc.)

**Notes: Multidisciplinary care**: Essential for managing WFS and related disorders, involving collaboration among specialists in various fields to provide comprehensive care and support

**Regular monitoring and Adjustments**: Important to address the evolving needs of the patient over time

### Novel therapeutic approaches

The main goal of disease-specific treatment in WFS should be to arrest the progression of the disease in all involved tissues. Any pathology-specific therapies should therefore prevent cellular aging and degenerative processes by preserving ER function, calcium homeostasis, protein folding and regulation of oxidation-reduction processes, the alterations which underlie the disease [43, 45, 49]. Ongoing research projects in regenerative medicine and gene therapy show promising results, although they have not yet reached the stage of clinical application, both for WFS and other neurodegenerative disorders [50]. Currently, the most promising therapeutic approach appears to be the use of chaperones, which play a crucial role in protein folding mechanisms within the ER. Ongoing trials are investigating the effectiveness of 4-phenylbutyric acid (PBA) and tauroursodesoxycholic acid (TUDCA). These compounds have demonstrated the ability to preserve β cell function by reducing ER stress and cell apoptosis, while also mitigating neurodegenerative processes [51]. Another therapeutic approach under investigation involves preventing calcium-mediated ER degeneration, and consequently cell apoptosis, by modulating cytoplasmic calcium homeostasis mechanisms. Dantrolene, already in use for some neurological diseases, acts through the suppression of calcium efflux from the ER to the cytoplasm, thus safeguarding β cell and neuronal integrity [52]. Drugs that bind the calcium-dependent ATP of the ER, Wolframin’s substrate, or inositol 3-phosphate-activated calcium channel receptors would exert a similar effect [53]. Valproic acid is currently under investigation as a novel therapy for preventing degenerative processes in neurons and β cells affected by WFS1. Acting as a neuroprotectant, valproic acid is the subject of a phase 2 clinical study recently initiated. In addition to these experimental clinical trials, several hypoglycemic agents already approved for diabetic treatment have demonstrated efficacy in managing glycemia in these patients, with, however, limited, and controversial effects on cell degeneration processes [50]. These drugs appear primarily effective in glucose homeostasis but do not target pathogenetic mechanisms of WFS1. The positive effects of GLP-1 receptor agonists (GLP-1Ras) and their efficacy in the treatment of type 2 diabetes prompted researchers to evaluate their efficacy in animal models to assess their therapeutic potential in the treatment of diabetes in WFS1. All GLP-1RAs evaluated, such as exenatide, liraglutide, and dulaglutide, improved glycemic control in both rodents and patients [54]. This efficacy may stem from the GLP-1 receptor’s modulation of the ER response, facilitating β cell adaptation and preventing apoptosis. However, diabetes represents just one side of the syndrome, as nearly all patients also experience neurological impairment, worsening the prognosis. Some data suggests that GLP-1Ras may reduce neuronal inflammation, thereby improving neurological prognosis. This evidence is supported by studies indicating that certain drugs, such as liraglutide and exendin-4, penetrate the blood-brain barrier and exhibit protective effects on optic nerve trophism, thus potentially preserving visual acuity in animal models and patients with WFS1 [55]. Moreover, WFS1 is associated with chronic inflammatory condition, which GLP-1RAs may mitigate by modulating immune system activity and reducing the production of proinflammatory cytokines. In conclusion, literature data suggest that GLP-1RAs could be effectively used as therapeutic agents in WFS1, not only improving glycemic control but also potentially delaying and slowing the onset of neurological complications. Future clinical studies are warranted to corroborate these effects and to evaluate potential synergies between GLP-1R, GIPR and glucagon receptor agonists, which have shown promising preliminary data in cellular and animal models of neurodegenerative diseases [54, 56].

### Non-pharmacological interventions

Since pharmacological treatments mainly focus on alleviating specific symptoms, there is a need for non-pharmacological approaches to effectively address the holistic and multisystemic nature of WFS. These approaches target key issues such as mobility, communication, sensory loss, and overall quality of life. Non-pharmacological interventions—including nutritional management, visual and hearing support, psychological and emotional care, physical therapy, educational modifications, and lifestyle changes—are essential for managing symptoms and enhancing the daily lives of affected individuals (as detailed in Table 6). Given the variability of WFS among patients, it is important to develop a personalized care plan that reflects everyone’s unique needs in collaboration with healthcare providers. This plan should be regularly updated as the patient’s condition changes. Additionally, effective communication among healthcare providers, patients, and their families is crucial for successful care.

**Table 6:** Non-pharmacological interventions for management of Wolfram syndrome

Intervention	Symptom/problem/need	Indication
Nutritional support	Impaired glucose homeostasis	• Careful dietary management is crucial. Collaborating with a dietitian to plan carbohydrate intake can help stabilize blood sugar levels in line with insulin regimens
	Excessive thirst and urination	• Maintaining proper hydration and monitoring electrolyte levels is essential; adjust dietary intake as necessary
Visual support	Progressive vision loss	• Low vision aids, such as magnifiers, specialized lighting, and large-print materials, can help maximize remaining sight • Technology like screen readers and audiobooks enables continued reading and access to information • Orientation and mobility training promotes safe navigation for patients with vision impairment, enhancing independence
Hearing support	Hearing loss	• Hearing aids can significantly improve communication for patients with mild to moderate hearing loss by amplifying sounds. • Cochlear implants may be an option for those with severe hearing loss to restore some hearing capability • Speech therapy and learning sign language can enhance communication effectiveness
Emotional and Psychological support	Psychological stress	• Regular counseling or therapy sessions can help manage anxiety, depression, and stress associated with chronic illness • Techniques such as mindfulness, meditation, and relaxation exercises can alleviate stress and emotional challenges • Support groups can provide emotional assistance by allowing patients and families to share experiences and advice
Educational support	Learning problems	• An individualized education plan tailored to specific needs can benefit children. This may include special education services, modified materials, and extended exam time • Depending on vision and hearing loss severity, patients might benefit from tools such as Braille materials or sign language interpreters
Physical and occupational therapy	Motor skills impairment	• Physical therapy can help maintain strength, balance, and coordination, essential for daily activities • Regular, adapted exercise can enhance overall health and well-being, focusing on safe and enjoyable activities considering the patient’s abilities • Occupational therapy assists patients in adapting daily tasks, making activities like dressing, eating, and writing easier and more manageable
Support to families and caregivers	Disease progression and changing needs	• Family members and caregivers should understand the progressive nature of the disease and how to provide appropriate support in daily care
Legal support	Medical and social needs	• Legal considerations for patients with Wolfram Syndrome should include: • Rights to access healthcare and educational support • Guardianship and decision-making authority for minors and those unable to care for themselves • Disability rights and accommodations in education and employment • Legal aspects of advanced care planning and end-of-life decisions

#### Patients’ associations

Living with a rare disorder like WFS can be both isolating and frustrating for patients and their families. Patient associations play a vital role in connecting individuals with similar experiences, creating a support network that offers comfort, alleviates feelings of isolation, and provides practical advice for managing daily challenges related to the condition. Numerous patient associations and institutions dedicated to WFS exist globally (see Table 7). By educating patients and their families, these organizations empower them to make informed decisions about their care. Strict cooperation with patients’ and families’ associations will increase knowledge of the disease among people and caregivers, sensitize health care institutions about patients’ needs and improve their quality of life. Furthermore, associations may provide a peer support among patients and caregivers.

**Table 7:** Patients’ associations and institutions focused on Wolfram syndrome

Location	Name	Website
Italy	Sindrome di Wolfram Italia - Associazione Gentian	www.sindromewolframitalia.com
United Kingdom	Wolfram Syndrome UK	www.wolframsyndrome.co.uk
France	Association du syndrome de Wolfram (ASW)	https://endo-ern.eu/patient-organisation/association-du-syndrome-de-wolfram-asw/
United States	Worldwide Society of Wolfram Syndrome Families	www.wolframsyndrome.org
	Snow Foundation	https://thesnowfoundation.org/
	The Ellie White Foundation for Rare Genetic Disorders	https://elliewhitefoundation.org/
	Washington University Wolfram Syndrome International Registry & Clinical Study	https://wolframsyndrome.wustl.edu/
Spain	Asociación Española para Investigación y Ayuda al Síndrome de Wolfram	https://aswolfram.org/
Germany	Wolfram Syndrome Deutschland	https://wolfram-syndrom.de/
Brasil	Associação Brasileira da Síndrome de Wolfram (ABSW)	https://www.wolframinside.org/absw/

## Prognosis and follow-up

The symptoms of WFS significantly impair patients’ quality of life and daily activities [57, 58]. The lifespan of individuals with WFS typically ranges from 30 to 40 years. The most frequent causes of death include hypoglycemic coma, status epilepticus, end-stage renal disease from recurrent urinary tract infections, central respiratory failure associated with brainstem atrophy, and suicide. Current life expectancy for individuals with WFS is improved compared to past reports [6]. Observation from a national specialized multidisciplinary clinical team, which followed 40 affected adults, showed that the average age of the patients was 37 years, with the oldest individual being 65 years old [59]. Following the initial diagnosis of WFS, a comprehensive assessment of comorbidities is required. For detailed timing and specific assessment measures, refer to Table 8.

**Table 8:** Comprehensive assessment plan for Wolfram syndrome comorbidities

Condition	Initial diagnosis	Follow-up
Insulin-dependent DM	- Diagnosis per ISPAD 2022 guidelines - Mixed meal tolerance test for C-peptide secretion	- Confirmed: Outpatient evaluation every 3–4 months or based on glucometrics - Unconfirmed: Annual assessment
Optic nerve and retinal atrophy	- Complete neuro-ophthalmological exam - Ishihara test, visual field, OCT, OCT-A, visual evoked potentials, pERG, fundus evaluation, optic tract MRI	Annual follow-up with the mentioned examinations
Sensorineural Hearing Loss (SHL)	- Audiogram - Auditory evoked potentials	Follow-up with the same examinations
Neurological dysfunction	- Neurological exam including cranial nerve assessment, brain MRI	Annual follow-up with the same examinations
Respiratory dysfunction	- Polysomnogram	Annual follow-up with polysomnogram when evidence of brainstem atrophy at MRI.
Psychological assessment	- Support for patients and caregivers to reduce the distress, with particular attention to emotional needs, adaptation process and psychological risk factors	Annual follow-up with particular attention to quality of life and emotional needs and treatment burden
Psychiatric symptoms	- Assessment test for anxiety-depressive symptoms	Annual follow-up with the same assessment
Urological dysfunction	- Evaluation of urinary tract infection frequency, urinary urgency, renal function, urinary echo with post-mineral residual, possible urodynamic exam	Annual follow-up with the mentioned evaluations
Other endocrinological manifestations	-Diabetes Insipidus: Weekly water balance, plasma/urinary osmolarity, sodiemia, copeptin (if available) - Hypothyroidism: TSH, fT4 - Hypogonadism: Pubertal progression, LH, FSH, testosterone or oestradiol, AMH, Inhibin B - Hyposomia/Growth Velocity Impairment: Growth curve evaluation, GH stimulation test	Annual follow-up with respective tests

Notes: **Multidisciplinary approach**: Management and follow-up of WFS require a comprehensive and coordinated approach involving various specialties

**Individualized care**: Follow-up intervals and diagnostic tests are tailored based on the individual’s condition and response to treatment

**Early detection and continuous monitoring**: Essential for managing the progression of WFS and its comorbidities effectively

## Transition

The transition phase refers to the process of moving patients from pediatric to adult healthcare services. This sensitive period requires coordinated planning and management to ensure that adolescent patients continue receiving appropriate, effective, and comprehensive care as they shift from child-oriented to adult-oriented healthcare systems. Given the complexity of the disease, transitioning individuals with WFS, diagnosed in adolescence or even childhood, demands a multidisciplinary approach to ensure continuity of care and address both medical and psychosocial needs. Currently, there is no literature available on this topic in relation to WFS. However, lifelong surveillance and multidisciplinary care are essential cornerstones in the management of this syndrome, as they are for most rare diseases. There is no fixed age for transition; it should be planned based on the individual’s maturity, psychological status, cognitive abilities, long-term care needs, social and personal circumstances, and communication requirements [60]. The transition to adult care also involves significant psychosocial and educational challenges. Therefore, psychosocial support and educational planning are pivotal. Patients and their families may experience anxiety about the future and adapting to new healthcare settings. Providing psychological support and counseling is crucial to help them manage these changes and improve their overall well-being. As patients enter adulthood, it is equally important to support their educational and vocational development. Personalized plans that align with the patient’s abilities and interests can enhance their quality of life and foster greater independence. To ensure an effective transition, the following recommendations are essential:

1. Early Planning: Begin transition planning well in advance, ideally 1–2 years prior. Early preparation allows for a smoother adjustment to adult care services and helps address the patient’s evolving needs.
2. Individualized Care Plans: Develop personalized transition plans that address the specific medical, psychosocial, and vocational needs of each patient. These plans should be regularly reviewed and updated to reflect changes in the patient’s condition or circumstances.
3. Family Involvement: Actively include family members in the transition process. Supporting and informing parents and caregivers is essential to help them adapt and continue providing effective care for the patient.
4. Collaborative Approach: Foster seamless communication and collaboration between pediatric and adult care teams. Ensuring accurate documentation of the patient’s medical history and treatment plans is crucial to maintaining continuity of care.

We recommend that the transition phase be overseen by the pediatrician or pediatric diabetologist. A follow-up meeting to review the shift to adult-oriented healthcare is suggested after the transition, with a timeline personalized to the patient’s individual characteristics and needs. We believe that by addressing these factors, the transition experience for individuals with WFS can be significantly improved, leading to better quality of life and long-term outcomes, while avoiding any gaps in medical care.

## Conclusion

This expert consensus provides a comprehensive framework for elevating the standard of care for individuals with WFS. By leveraging the collective knowledge of specialists across disciplines, the document underscores the critical role of early genetic identification and a thorough, multidisciplinary diagnostic approach in managing WFS. It advocates for personalized management strategies that address the multifaceted needs of patients with WFS, while also acknowledging the promising prospects of novel treatments, albeit with a measured view of their present developmental phase. Moreover, this consensus highlights the imperative for continuous education among healthcare providers. Enhancing the competence of medical professionals in recognizing, understanding, and treating WFS is depicted as a cornerstone for improving patient outcomes. Knowledge of WFS has increased over the last year, improving healthcare professional clinical practice and leading to more favorable patient experiences and prognoses. This expert consensus advocates for a systematic and evidence-informed approach, summarizing advancements in the early detection, therapeutic intervention, and comprehensive care of WFS. It encourages concerted research and clinical initiatives aimed at refining and broadening the scope of care strategies for WFS. Hopefully, the ongoing research will provide significant insights into physiopathology of this syndrome, providing significant breakthroughs in patient care and treatment efficacy.

## Electronic supplementary material

Below is the link to the electronic supplementary material.

Supplementary Material 1

Supplementary Material 2
