# Supplementary material for: SID/SIEDP expert consensus on optimizing clinical strategies for early detection and management of wolfram syndrome
Source: J Endocrinol Invest. 2024 Nov 11;48(3):507–25. doi: 10.1007/s40618-024-02495-z (PMC11876246; doi:10.1007/s40618-024-02495-z)
Supplement: Supplementary file 1 — Supplementary Material 1 [file 40618_2024_2495_MOESM1_ESM.pptx]

## Slide 1
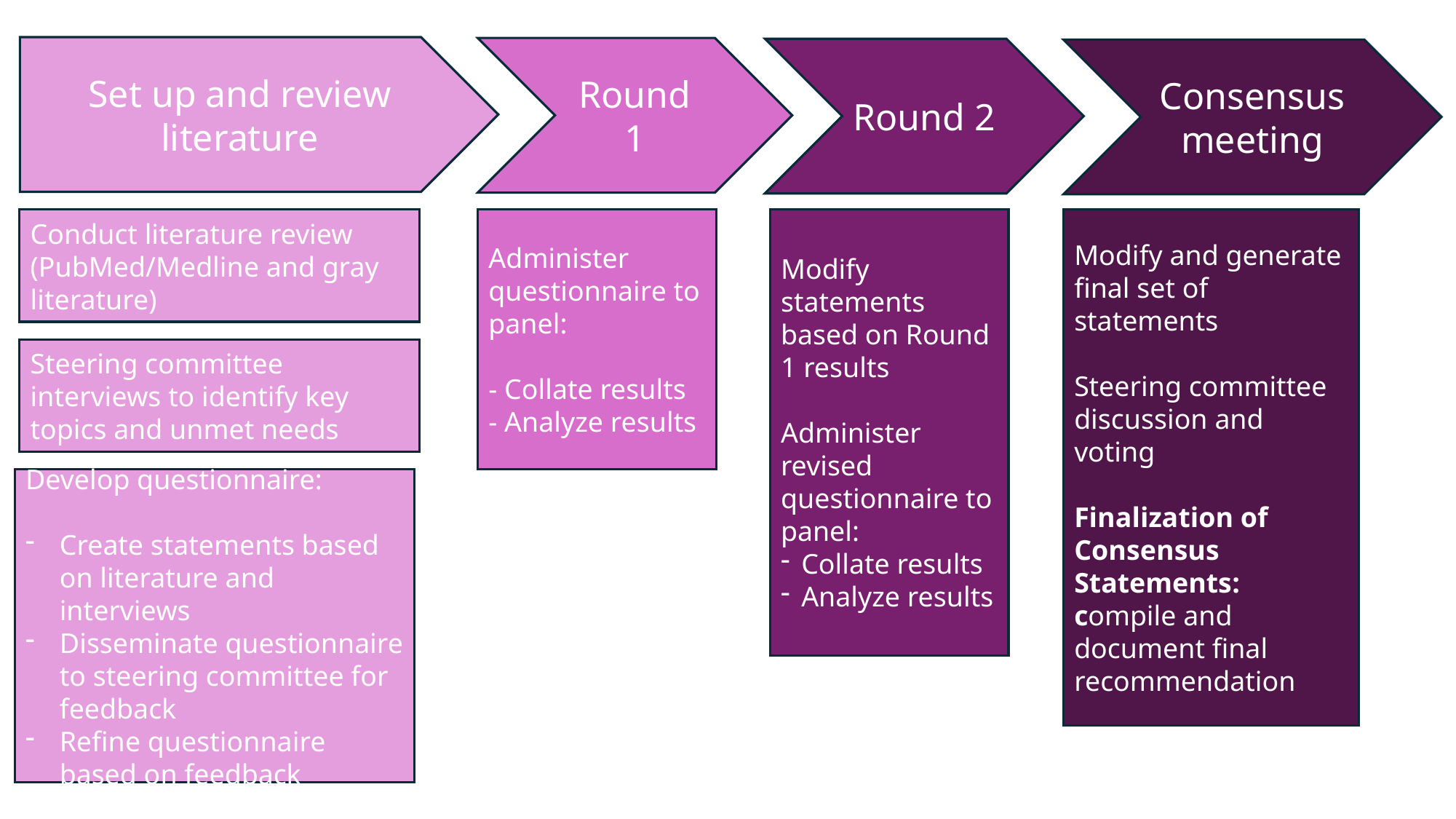

Set up and review literature
Round 1
Round 2
Consensus meeting
Modify statements based on Round 1 results
Administer revised questionnaire to panel:
Collate results
Analyze results
Modify and generate final set of statements
Steering committee discussion and voting
Finalization of Consensus Statements:
compile and document final recommendation
Administer questionnaire to panel:
- Collate results
- Analyze results
Conduct literature review (PubMed/Medline and gray literature)
Steering committee interviews to identify key topics and unmet needs
Develop questionnaire:
Create statements based on literature and interviews
Disseminate questionnaire to steering committee for feedback
Refine questionnaire based on feedback
