# Supplementary material for: SID/SIEDP expert consensus on optimizing clinical strategies for early detection and management of wolfram syndrome
Source: J Endocrinol Invest. 2024 Nov 11;48(3):507–25. doi: 10.1007/s40618-024-02495-z (PMC11876246; doi:10.1007/s40618-024-02495-z)
Supplement: Supplementary file 2 — Supplementary Material 2 [file 40618_2024_2495_MOESM2_ESM.docx]

**Supplemental Table 1. Delphi survey (Round 2)**

|  | **Topics** | **Statements** | **Consensus >75%** | |
| --- | --- | --- | --- | --- |
|  |  |  | **Yes** | **No** |
| **1** | **Scope and Aims** | 1.1. This document is designed for healthcare professionals at all levels, aiming to improve the diagnosis, treatment, and management of WFS patients, emphasizing the necessity of services across various specialties (see Table 1) for comprehensive care. | X |  |
|  |  | 1.2. The consensus seeks to standardize care for WFS patients, ensuring equitable access to diagnosis, treatment, and follow-up, thus enhancing healthcare efficiency and addressing variability in clinical decisions. | X |  |
| **2** | **Unmet Needs** | 2.1. Early Diagnosis and Genetic Testing: Due to genetic diversity and varied clinical manifestations, diagnosing WFS is challenging. Connecting disparate symptoms to identify WFS as the underlying condition poses difficulties for patients and providers. Access to genetic testing for timely diagnosis and management is often limited, especially in underserved regions. | X |  |
|  |  | 2.2. Targeted Treatments: Currently, no treatments specifically target the underlying genetic cause of WFS; existing therapies mainly address symptoms (e.g., insulin for diabetes). Research into disease-modifying treatments or gene therapies is ongoing. | X |  |
|  |  | 2.3. Comprehensive Diagnosis and Appropriate Care: The complexity of the disease leads to delays in achieving a comprehensive diagnosis and appropriate care, which can hinder timely treatment initiation and worsen disease progression. | X |  |
|  |  | 2.4. Specialized Clinics: Limited specialized centers for rare disorders like WFS restrict access to optimal care and research trials. Comprehensive multidisciplinary evaluations and treatments are necessary as WFS affects multiple body systems over time. | X |  |
|  |  | 2.5. Medical Community Awareness: Due to its rarity, many healthcare professionals are unaware of Wolfram Syndrome, which results in delays in diagnosis and care. Increasing awareness through training and continuing education programs is vital. | X |  |
|  |  | 2.6. Effective Guidelines: A collaborative, multidisciplinary approach is essential for managing WFS. | X |  |
|  |  | 2.7. Non-Pharmacological Interventions: The progressive nature of WFS and its impact on various systems create significant social, emotional, and psychological challenges for patients and their families, which are often under-recognized but crucial for overall well-being. | X |  |
| **3** | **Clinical presentation** | 3.1. WFS is a rare neurodegenerative disease with juvenile onset, primarily characterized by diabetes mellitus (DM), optic atrophy (OA), diabetes insipidus (DI), and deafness (D), collectively known as DIDMOAD. | X |  |
|  |  | 3.2. Additional clinical features may include urinary tract, endocrinological, renal, psychiatric, and neurological abnormalities (see Table 3). | X |  |
|  |  | 3.3. WFS is a condition characterized by significant clinical and genetic diversity. To date, two distinct forms of WFS have been identified, both of which are inherited in an autosomal recessive manner. WFS type 1 (WFS1, OMIM #222300) is the most common form, resulting from biallelic mutations in the wolframin gene (WFS1). WFS type 2 (WFS2, OMIM #604928) is much rarer and is caused by biallelic mutations in the CISD2 gene. Clinical manifestations of WFS2 can include upper intestinal ulcers, mucocutaneous bleeding, and impaired platelet aggregation. | X |  |
|  |  | 3.4. *WFS1*-related conditions presenting with autosomal dominant inheritance due to heterozygous WFS1 mutations have also been reported (see Table 2) | X |  |
|  |  | 3.5. WFSL can present with a variable combination of clinical manifestations, including OA, DM, and other symptoms. The most common phenotype is OA and hearing impairment, seen in 47% of patients. DM occurs in 44%, and cataracts are also common. Typically, hearing impairment is the first manifestation, followed by DM and OA. | X |  |
|  |  | 3.6 Published follow-up data on WFSL are scarce. Existing data indicate that WFSL presents as a non-progressive clinical condition with a milder phenotype compared to classical WFS. Individuals affected by WFSL do not seem to experience progressive neurodegeneration; however, additional neuroradiological studies are needed to confirm this finding. | X |  |
| **4** | **Prevalence** | 4.1. WFS is estimated to affect about 1 in 160,000 to 770,000 individuals, with prevalence varying by region: in North America, it is estimated at 1 in 100,000, while in the UK it is 1 in 770,000. Recent estimates indicate 1 in 54,478 in Messina, northeastern Sicily, 1 in 805,000 in Northern India, and 1 in 1,351,000 in Italy. | X |  |
| **5** | **Diagnostic Criteria** | 5.1. Clinical suspicion of WFS should arise with the presence of two main findings: insulin-dependent diabetes mellitus with onset before age 15 and early-onset optic atrophy. | X |  |
|  |  | 5.2. Additional signs and symptoms relevant for WFS diagnosis include central diabetes insipidus (usually diagnosed in the second decade) and sensorineural deafness (typically identified around age 16 in 60% of cases). | X |  |
| **6** | **Differential Diagnosis** | 6.1. The differential diagnosis between WFS1 and WFS2 can be established clinically based on observed signs and symptoms, with molecular confirmation via genetic testing for the WFS1 and CISD2 genes. | X |  |
|  |  | 6.2. Some symptoms of WFS may overlap with other genetic conditions; special attention is required to distinguish WFS from conditions featuring syndromic hearing loss, ocular defects, and neurological abnormalities, with or without diabetes (see Table 4). | X |  |
| **7** | **Prognosis** | 7.1. The prognosis of WFS is primarily associated with the severity of neurological symptoms, which is generally poor. Most patients experience premature death due to severe disabilities, with a median age of death around 30 years (25-49 years). | X |  |
| **8** | **Comprehensive care of patients** | 8.1. Various specialties are involved in the diagnosis and care of patients, as listed in Table 1. | X |  |
|  |  | 8.2. Several comorbidities are observed in WFS, which should be diagnosed and managed as detailed in Table 6. | X |  |
| **9** | **Genetic Testing** | 9.1. Molecular screening for WFS should include two genes: WFS1 and CISD2 to confirm diagnosis. | X |  |
|  |  | 9.2. Molecular screening is essential to identify causative mutations and confirm the clinical diagnosis. This screening should be performed in specialized diagnostic centers utilizing Next Generation Sequencing (NGS) techniques, including targeted NGS panels, whole exome/genome sequencing, and MLPA, to accurately confirm a WFS diagnosis. Whenever feasible, molecular screening should also be extended to the parents and first-degree relatives of the probands. | X |  |
|  |  | 9.3. Genetic variants must be accurately classified and reported using the five-category system validated by the ACMG and AMP. Only pathogenic and likely pathogenic variants should be reported; variants of uncertain significance (VUS) should be monitored for possible reclassification. Genetic testing results should be communicated in a comprehensive counseling session involving a multidisciplinary team. | X |  |
|  |  | 9.4. Genetic testing is valuable for diagnosing and managing WFS, as it helps confirm clinical diagnoses, identify carriers, plan genetic counseling, and monitor disease progression based on genotype-phenotype correlations. | X |  |
|  |  | 9.5. Genetic counseling is necessary to provide detailed information about the significance and limitations of tests. It should consist of pre-test and post-test counseling sessions. | X |  |
|  |  | 9.6. Given the progressive nature of WFS, results from genetic testing should be communicated in a comprehensive counseling session, facilitated by a multidisciplinary team including a geneticist and other clinical specialists involved in disease management | X |  |
| **10** | **Genotype-phenotype correlations** | 10.1. Recent research by Lee et al. provides information on genotype-phenotype correlations, which can help clinicians accurately assess the severity of WFS and aid in predicting prognosis and tailoring personalized treatments. | X |  |
| **11** | **Treatment** | 11.1. Currently, there is no specific treatment for WFS; each associated disorder can be managed with hormone replacement therapies or palliative care (see Table 5). | X |  |
|  |  | 11.2. Current treatment approaches do not address the underlying cause, focusing instead on clinical monitoring and supportive care to mitigate debilitating effects. | X |  |
|  |  | 11.3. No therapies exist to prevent the ongoing deterioration of the condition, as they do not target the underlying cause. | X |  |
|  |  | 11.4. Novel therapeutic approaches may include regenerative medicine, gene therapy, chaperones, preventing calcium-mediated endoplasmic reticulum degeneration, drugs targeting calcium-dependent ATP of the ER, and GLP-1 receptor agonists for improving glycemic control and potentially delaying neurological complications. | X |  |
|  |  | 11.5. Non-pharmacological approaches are necessary to address the holistic and multisystemic aspects of WFS, focusing on mobility, communication, sensory loss, and overall quality of life. | X |  |
|  |  | 11.6. Non-pharmacological interventions should be include all those listed and detailed in Table 7. | X |  |
| **12** | **Transition** | 12.1 Given the complexity of the disease, transitioning individuals with WFS, diagnosed in adolescence or even childhood, demands a multidisciplinary approach to ensure continuity of care and address both medical and psychosocial needs | X |  |
|  |  | 12.2 There is no fixed age for transition; it should be planned based on the individual’s maturity, psychological status, cognitive abilities, long-term care needs, social and personal circumstances, and communication requirements | X |  |
|  |  | 12.3 Successful transitions should begin 1-2 years in advance to facilitate a smooth adjustment to adult care and address the patient’s evolving needs. Individualized care plans are essential, tailored to each patient’s medical, psychosocial, and vocational requirements, with regular updates to reflect any changes. | X |  |
|  |  | 12.4 Engaging family members actively in the process is crucial, as it provides them with the support and information needed to continue effective care. Additionally, promoting strong communication and collaboration between pediatric and adult care teams is key to maintaining continuity, supported by accurate documentation of the patient’s medical history and treatment plans. | X |  |
